# An analysis of free-choice electives in an undergraduate medical degree

**DOI:** 10.1186/s12909-017-0955-7

**Published:** 2017-07-11

**Authors:** Catriona Daly, Jason Last

**Affiliations:** UCD School of Medicine, University College Dublin, Belfield, Dublin 4 Ireland

**Keywords:** Medical students, Medical school, Undergraduate, Elective programme, Elective choices, Options, Modules, Student motives, Assessment strategy

## Abstract

**Background:**

The University College Dublin Elective Programme was introduced in 2005 with the intention of broadening the learning of its undergraduate students. Undergraduate medical students undertake seven free-choice electives during their six-year degree programme. They are permitted to choose electives from any school in the University. Students also have the option of selecting ‘In-Programme’ electives, which are aligned to medical themes. The purpose of this study is to analyse the electives taken by medical students with a view to better understanding the factors that influence their choices.

**Methods:**

In this mixed methodology study, the quantitative phase consisted of a retrospective analysis of 3318 elective choices associated with 474 medical students between 2006 and 2013. Elective choices were analysed in terms of popularity, difficulty level and subject matter. The prospective qualitative phase consisted of a series of semi-structured focus groups held with current medical students. Discussions from the focus groups underwent thematic analysis with a few to exploring and clarifying the quantitative findings.

**Results:**

The most frequently chosen electives were In-Programme (38.6%) and Applied Language (21.6%) electives, with patterns not significantly varying from year to year. Male and female students tended to take the same type of electives. Focus group discussions revealed that the primary factor motivating choice was workload, with students preferring less demanding electives. Participants indicated that elective grading and assessment criteria had a significant impact on their choices. Participants described ways in which the elective selection process could be improved, including a desire for more structured electives and a revision of the elective selection process.

**Conclusions:**

The retrospective data analysis revealed a high level of consistency in medical students’ elective choices from stage to stage and between different year cohorts. Qualitative investigation revealed that medical students tend to focus on grading, assessment strategies and skills development when choosing their electives. The implication that students may be reluctant to take more challenging electives despite having an interest in the subject is one that warrants consideration when designing or adapting programmes for the future. Although these findings are associated with a free-choice elective programme, similar strategies are likely to be employed for the more traditional option-based programmes that are associated with the majority of medical degrees internationally.

## Background

The University College Dublin (UCD) Elective Programme was introduced to undergraduate bachelor degree courses in UCD in September 2005 [1, 2]. Under this programme, students are obliged to complete twelve modules per academic year, comprised of ten ‘core’ or ‘option’ modules that are specific to the degree course, and two ‘elective’ modules that can be chosen from any department in the University. Within certain practical limitations, students can choose as their elective any module offered within any school in UCD. Students also have the option of taking ‘In-Programme’ electives, which are tailored to specific courses and to which students in that course have priority [1, 2].

The UCD Elective Programme is based on a philosophy of education attributed to John Henry Cardinal Newman, who founded UCD in 1854. In his book ‘The Idea of a University’ Newman [3] outlined his beliefs that a university education should be broad rather than specialised; he emphasised that “*a university should…be open to teaching anything that is knowable*”, and he favoured, “*the education of the whole mind*”, placing a specific value on “*learning for its own sake*”.

The purpose of the UCD Elective Programme is to establish a student-centred approach to education by enabling undergraduate students to broaden their learning across different academic fields and to deepen their knowledge within their chosen field as well as enhancing their academic skillset and encouraging them to pursue their interests [1, 2].

Giving students a wide range of choice has been shown to promote interest and enthusiasm in their studies and this has been demonstrated repeatedly to improve quality of learning [4, 5]. Allowing students to choose some of their modules each semester is a vital tool for maintaining and nurturing interest [5] as well as developing personal skills that may otherwise be neglected in a purely exam-focused discipline [6]. A survey carried out in 1991 by the Northeast Missouri State University indicated that, in general, students like the idea of free-choice electives and would welcome more of them in their degree programme [7].

In medical education in particular, the idea of self-selected electives is one that has been strongly promoted by bodies such as the General Medical Council (GMC) [6] and the Association for Medical Education in Europe (AMEE) [8]. The GMC advocates the effectiveness of optional electives as a mechanism to address the well-recognised issue of ‘information overload’ associated with medical education [9]. Information overload refers to the extensive volume of information relayed during the process of medical education, the nature of which has been shown to impact negatively on the student and consequently on the emerging doctor [6, 9]. The GMC recognised this issue as far back as 1863 [6] and numerous efforts have been made since then to move away from a purely didactic teaching method, a process which is regarded as ‘dehumanizing’ [10, 11] and which promotes the passive acquisition of knowledge that may be outdated by the time the student graduates [6]. One such effort was the recommendation by the GMC in 1993 to introduce a core vs special study component in medical teaching programmes. Similarly, an all-elective fourth year was trialled in some medical schools in the USA, including the University of Kentucky in 1968–69 [12] and the University of Michigan in 1970–71 [13]. However, it should be noted that, in contrast to the Elective Programme in UCD, these were clinical or medical research electives rather than liberal (free-choice) electives.

Investigations into the outcome of these initiatives focused primarily on the observed benefits and career outcome for the students in question [14]. Few analyses inquired into the pattern of student choices, although this may be attributed to the fact that there were limited choices in the first place and that the introduction of core versus special study components has never been uniformly implemented across universities [6].

A literature review revealed substantial volumes of research relating to the academic decisions made by university students, including the reasons behind their choices, the potential problems arising from poor decision-making and the demographical variation in academic choice [5, 15–17]. Various factors have been demonstrated to influence these decisions, including interest in the subject matter, perceived difficulty of subject material and potential future career skills development [17]. It must be noted that the majority of this research involves the student body as a whole, with particular respect to the choice of major [15] and is not specific to medical students. The factors influencing the decisions of a medical student in selecting electives may vary significantly from those affecting the general student body, especially when considering the ‘information overload’ issue referred to previously [6]. That research which is specific to medical education appears to have focused on eventual career direction as opposed to elective choice as a student [13].

According to Mayo and Miciak [16], it is important to understand the reasons why student choose their electives in order to “*assist a university in curriculum design and in the allocation of space and teaching resources*”. A review by Ting and Lee [17] compiled a comprehensive list of potential influences as follows: level of interest, level of difficulty, timing of lectures, popularity/personality/leniency of instructors, expertise of instructors, grading and assessment format, exposure to career skills, influence of friends/family and average class size. Their study concluded that, among a group of marketing students in Malaysia, perceived difficulty of the module and perceived interest in the subject were the two most important factors in elective choice [17]. It is of interest therefore to assess whether medical students follow this trend or whether there is unique pattern in this group.

The Elective Programme is applied to UCD’s six-year undergraduate medical programme but not to its four-year graduate medical programme which is more aligned to many international models where students complete an undergraduate degree before starting medical school as a graduate, as in the United States [18]. The UCD graduate medical programme accepts applicants who have completed any four-year undergraduate degree. Although many of these graduate students will have completed a purely science-based or ‘pre-med’ degree specifically designed for those aiming for post-graduate medical studies, specific value can also be placed on alternative liberal arts or humanities undergraduate degrees [18], particularly as more liberal undergraduate degrees can be shown to improve students’ communication skills and cultural insight [18]. Indeed, the GMC emphasises the importance of academic diversity, saying that the constant advancement of medical sciences “*will inevitably become increasingly dependent on the ideas and techniques of other disciplines, such as mathematics, physics, philosophy and the social sciences*” [6]. A liberal elective programme such as that in UCD’s undergraduate medical degree embodies a rather different approach to integrating various disciplines into a single academic journey.

Undergraduate medical students in UCD undertake seven free-choice elective modules during the first three and a half years of their six-year course. All modules in UCD are assigned a level of difficulty. Level 1 is the equivalent difficulty of a stage 1 (first year) module, level 2 is the equivalent difficulty of a stage 2 (second year) module, and so on. Electives of any difficulty level may be selected so that students can choose either to widen their curriculum or to deepen their knowledge within particular disciplines. Every module has its own assessment, the outcome of which is a grade. Each grade is equivalent to a numerical value called a grade point, which ranges from 0 to 4.2. The average of the twelve grades achieved over the course of an academic year is calculated to produce the stage GPA (grade point average). In this way, the grade awarded in an elective module is given the same weighting as a grade awarded in any of the core modules. A ‘structured elective’ refers to a set of pedagogically linked modules in a certain subject area that may be taken as a block and acknowledged as an additional minor qualification on the student’s transcript.

The purpose of this study was to investigate the type of electives chosen by medical students in UCD and to determine whether or not a pattern may be observed. The GMC emphasises that medical curricula should provide options to enable students to design their own learning [6]. The UCD Elective Programme encompasses a fairly liberal approach to designing one’s own learning and therefore the analysis of how students select their electives may be viewed as a surrogate for what medical students consider to be important in their learning and what they would choose to prioritise. As alluded to previously, there is a gap in the literature with regard to liberal (free-choice) electives undertaken in a medical degree, which is why this project focused solely on medical students.

The information gathered was utilised to produce recommendations in order to optimise the benefits of the Elective Programme for future students in UCD. It was also designed to be of use to those designing any programme in which students have the power to design their own curriculum, whether that be through liberal electives, or as in the case of most medical programmes, through the use of a fixed number of optional modules.

## Methods

The methodology included two distinct phases, a retrospective quantitative phase and a prospective qualitative phase.

### Quantitative phase

Retrospective anonymised data was sourced from archived student records within the UCD School of Medicine. This information was stored on one device accessible only to the primary author, to be retained for a five-year period. The primary source data is permanently accessible through student records. The data obtained consisted of a list of the elective modules chosen by undergraduate medical students in UCD between September 2006 and September 2013 and also included student gender and the year of enrolment in the undergraduate medical degree programme. Information obtained on electives included level, subject, school and In-Programme or non-programme status as applied to medical students. The information was collated into an Excel spreadsheet which was then used to extract descriptive statistics and carry out the simple analyses required to establish trends associated with elective choice.

Each student was assigned a randomised number by which to identify the consecutive elective choices applying to that individual. Each undergraduate medical student chose seven electives over the first four years of the degree – two electives in each of stage 1, 2 and 3 and one elective in stage 4. The data was refined in order to establish a ‘cohort’ of students who had enrolled into the six-year undergraduate medical degree at UCD between 2006 and 2013 and who had completed seven electives during this time frame. Exclusion criteria were as follows: students who had advance entry (i.e. did not complete stage 1 due to prior academic performance); students enrolled in the degree programme prior to 2006; students who took electives after 2013; and students who failed to register for seven electives.

In total, 474 students were included in the study taken from five different year cohorts. A year cohort refers to a group of students who enrolled in the degree programme in the same academic year. The year cohorts were divided as follows: 86 students who started the undergraduate medical degree in September 2006; 99 who started in 2007; 96 who started in 2008; 101 who started in 2009; and 92 who started in 2010. Of these students, 209 were male and 265 were female. The data was reviewed within a series of Excel spreadsheets and analysed according to school offering the elective, elective difficulty level, year cohort, stage in the medical programme and student gender.

### Qualitative phase

Qualitative analysis was carried out by means of semi-structured focus groups held with current UCD undergraduate medical students who volunteered to take part anonymously. Convenience sampling was employed. Students were invited to participate via an open call e-mail distributed to stage 4 medical students and with a direct approach to students who were known to be completing on-campus research during this out-of-semester research period. The sample total was sixteen students.

The purpose of the focus groups was twofold: firstly, to investigate the motives driving elective choice among medical students; and secondly, to explore the attitudes of medical students towards the Elective Programme as a whole. The focus group discussions were loosely organised, with the moderator given a list of questions to discuss.

The initial pilot group consisted of a sample of students from stage 1 (having completed two electives) and from stage 4 (having completed all seven electives). The purpose of this pilot group was to help develop questions for the proceeding focus groups, based on methods used by Ting and Lee in their 2011 study entitled ‘Understanding Students’ Choice of Electives and its Implications’ [17]. Subsequently, two further focus groups were held with stage 4 undergraduate medical students. The total number of participants across the three focus groups was sixteen.

The discussions were recorded on a UCD-owned device and transcribed by the primary author before undergoing thematic analysis. Both authors reviewed the data to establish and agree on the major themes based on the frequency with which topics emerged and the degree of consensus among participants. The transcripts were then reviewed critically with respect to the major themes. Direct quotations of student participants were used in the thematic analysis.

The validity of the focus group findings was determined by the achievement of saturation. Saturation is here used to mean that findings were consistent from one focus group to the next and no new information pertinent to the research question was to be found by holding further focus groups. Sample credibility was assessed by comparing the qualitative findings to the quantitative findings, where consistency was found with regard to the popular elective categories. Having two separate reviews of the transcripts by both authors maximised confirmability of the findings. Dependability of the qualitative findings was limited by the use of convenience sampling and by the limitation of sampling to only one stage group. Although there is no reason to believe that students in other stages of the medical degree might have conflicting views, discussions with students from earlier stages in the programme might have contributed further to the findings, particularly as students in earlier stages would have been on the verge of selecting their own electives for the approaching semester.

## Results

### Quantitative phase

**Fig. 1:**
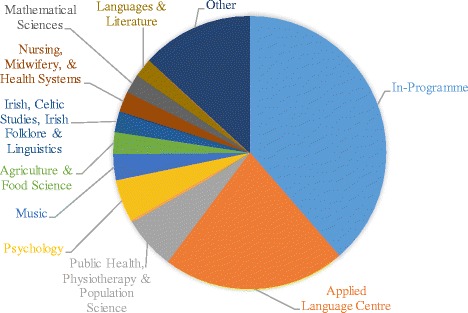
Pie chart to show popularity of elective categories

Initial results from the retrospective phase of investigation are shown in Table 1 and Fig. 1.

**Table 1:** Most popular elective categories by stage

	Stage 1	Stage 2	Stage 3	Stage 4	Total %	
In-programme	294	414	407	167	1282	38.64%
Applied language centre	250	206	181	80	717	21.61%
Public health, physiotherapy and population science	19	83	50	64	216	6.51%
Psychology	98	22	39	7	166	5.00%
Music	8	27	38	29	102	3.07%
Agriculture and food science	0	4	71	11	86	2.59%
Irish, celtic studies, Irish folklore and linguistics	33	28	15	7	83	2.50%
Nursing, midwifery and health systems	5	2	19	54	80	2.41%
Mathematical sciences	21	33	13	8	75	2.26%
Languages and literature	54	14	4	2	74	2.23%
Business	27	10	2	3	42	1.27%
Philosophy	8	15	7	2	32	0.96%
Other	131	90	102	40	363	10.94%

The most popular category of elective choice was ‘In-Programme’ (38.6% in total), a finding consistent across all stages and between all year cohorts. In-Programme electives were more frequently picked in stages 2 and 3 (43.7% and 42.9% respectively) than in stages 1 and 4 (31.0% and 35.2% respectively). Just under one third of the cohort (145 students) chose four or more In-Programme electives – representing more than half of their elective choices – two of whom (0.4%) chose only In-Programme electives. There were 31 students (6.5%) who chose not to take any In-Programme electives.

The second most popular category, ‘Applied Languages’, accounted for 21.6% of choices, although the popularity of these electives appeared to diminish from stage to stage, decreasing from 26.4% in stage 1 to 16.9% in stage 4. Nearly a quarter of the cohort (113 students) took three or more Applied Language electives. Interestingly, only four of these students (3.5%) continued with the same language throughout. The majority of these students (71.7%) took two or more introductory (difficulty level 1) Applied Language electives rather than progressing up through the levels. Of all the Applied Language electives taken, 56.5% were introductory modules. The most popular languages within the School of Applied Languages are shown in Table 2.

**Table 2:** Distribution of Applied Language electives

Language	Number of electives taken	
Spanish	277	38.6%
French	166	23.2%
Italian	109	15.2%
German	65	9.1%
Japanese	36	5.0%
Chinese	24	3.3%
Polish	13	1.8%
Swahili	12	1.7%
Arabic	6	0.8%
English for international students	4	0.6%
Russian	4	0.6%
Czech	1	0.1%

The next most popular elective categories were ‘Public Health, Physiotherapy and Population Science’ (6.5%), ‘Psychology’ (5.0%) and ‘Music’ (3.1%), as seen in Table 1.

**Fig. 2:**
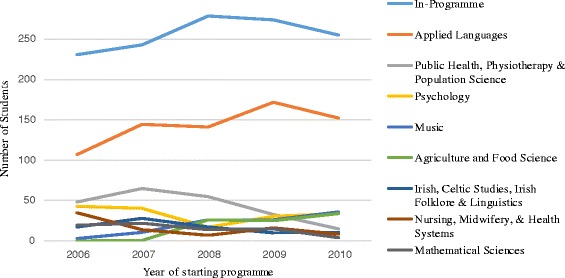
Graph to show trends in elective choice by year cohort

The patterns described above were replicated somewhat in the different year cohorts, with In-Programme electives and Applied Language electives being consistently the most popular categories. However, some elective categories such as ‘Agriculture and Food Science’ appeared to progressively gain popularity from 2006 to 2010, while others became less popular, such as ‘Public Health, Physiotherapy and Population Science’. This is shown in Fig. 2.

**Fig. 3:**
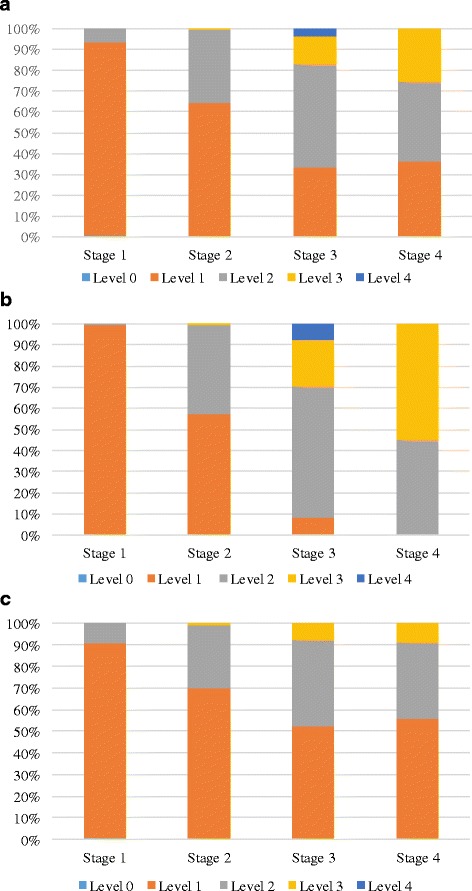
**a** Graph to show elective difficulty levels across stages. **b** Graph to show elective difficulty levels across stages of In-Programme electives only. **c** Graph to show elective difficulty levels across stages of non-programme electives only

The difficulty levels of electives chosen by students tended to increase with progressing stage as shown in Fig. 3. This trend was most prominent when examining the In-Programme electives in isolation, but was also evident in relation to non-programme electives.

**Fig. 4:**
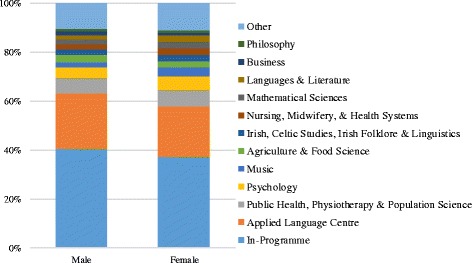
Graph to show elective choices of males and females by school

Figure 4 shows the comparison between the choices made by male and female students. Male students in the cohort appeared to be more likely to choose In-Programme and Applied Language Electives, however chi-square tests revealed no statistically significant differences between the choices made by male and female students.

The twenty most popular individual electives between 2006 and 2013 are listed in Table 3. Eight of the top twenty were In-Programme and a further six were Applied Language electives.

**Table 3:** Most Popular Electives

Elective Title	Type of elective	Number of students
Basic Principles of Trauma	In-Programme	251
Forensic Anthropology	In-Programme	210
Food Diet and Health	In-Programme	178
Introduction to Psychology for Healthcare	In-Programme	171
Spanish General Purpose I	Applied Language	169
Clinical Illustrations	In-Programme	159
Introduction to Massage	Public Health, Physiotherapy and Population Science	100
Italian General Purpose I	Applied Language	91
French General Purpose IV	Applied Language	81
Food Diet and Health II	Agriculture and Food Science	80
Multimedia Studies	In-Programme	68
Stem Cells in Medicine	In-Programme	60
UCD Symphony Orchestra	Music	57
Spanish General Purpose II	Applied Language	53
Psychology of Perception	Psychology	44
French General Purpose V	Applied Language	43
Introduction to Applied Psychology	Psychology	40
Introduction to Psychology	Psychology	35
French Language Ib	Applied Language	32
Rare Genetic Disorders	In-Programme	32

In summary, findings from the quantitative phase of the study indicate that there is a certain level of consistency in the choices made by medical students from stage to stage and between year cohorts and genders, with all groups favouring In-Programme electives followed by Applied Language electives.

### Qualitative phase

Thematic analysis of the qualitative data revealed a high level of consistency between each of the three separate focus groups. Findings from the focus groups are divided into two categories, the first being motives driving elective choice, and the second being general attitudes of students towards the Elective Programme as a whole.

### Motives driving elective selection

The broad themes surrounding elective selection were grading, assessment methods, interest and skills accumulation.

#### Grading

Invariably, participants reported that the most important factor when choosing electives was the likely grade that could be achieved in the elective, or in the words of one participant, “*which is easiest to get an A plus in*”. Participants emphasised that although the subject matter of their elective would not necessarily be relevant in their future medical studies, the grade achieved would reflect on their overall grade point average (GPA). It was thus acknowledged that the importance placed on grading was “*potentially a barrier to actually doing something that was interesting*”. Conversely, participants also recognised the appeal of choosing a less challenging elective purely for this purpose; “*if your GPA was low you might choose an easy elective to boost it*”.

Coinciding with the concerns about grading was the perceived workload of the elective, or the “*time commitment*” required. As one participant said, “*it’s very hard to justify putting a whole load of effort into a difficult elective and getting a bad grade, where you could have done an easy elective and done maybe an hour of revision… I’m not going to risk it*”. Another participant referred to “*the effort/reward ratio*” stating that “*if you can get an A but it’s going to take as much time as one of your core modules, then it’s just not going to happen*”.

It was apparent that a typically desirable (or ‘easy’) elective would be one with a low workload that is likely to yield a high grade. Participants did not refer to certain subjects as being easy or hard. They also made no reference to the assigned elective difficulty levels that are represented in Fig. 3. Instead they tended to refer to the assessment criteria with regard to whether a high grade could be achieved with an acceptably low level of time input. For all participants the primary source of information about the workload in different electives was “*recommendations from friends and older students*”, for example, “*if someone suggested a module that was easy*”.

#### Assessment strategy

The assessment strategy for an elective was considered an important factor by all participants. Participants generally preferred electives that had “*continuous assessment*” or “*split assessment*” as opposed to an “*end-of-semester exam that was worth 100% or… just an essay… that’s worth 100% of the marks*”. A number of participants expressed enthusiasm for one particular In-Programme elective, which was categorised as ‘GPA neutral’, meaning that the grade achieved in that elective did not contribute to the overall GPA. In this elective, students were awarded either a pass grade or a fail grade based on continuous assessment, and the elective was omitted from the total GPA calculation. Participants reported that this “*made the elective more enjoyable*” and “*much less time-consuming*”, even though they “*still had to turn up to classes and submit the assignments*”. One participant commented that “*there’s something to be said for making all electives GPA neutral*”, a sentiment that was echoed emphatically across all discussion groups.

#### Interest

Personal interest in the elective subject matter was a notion that persisted to some extent throughout each of the focus groups. Participants thought “*it was nice that they got the chance to do electives, and still get to learn about some of their interests*”, recognising the benefits of having “*the opportunity, without pressure, to explore other subject areas*”. In general, participants enjoyed doing electives, and appreciated the “*change of pace*”, describing liberal (free-choice) electives as a “*breather*” and “*an escape*” from the intensity of the core medical modules.

However, as previously suggested, there was a definitive barrier to students choosing these “*interesting*” electives in the form of GPA implications. As one participant said, “*just hearing that an elective wasn’t easy would already turn me off even if it sounded really interesting*”. Another participant admitted “*I didn’t get as much out of the Elective Programme as I should have*”, saying “*no matter how much interest you have in something, if it’s too hard you’re not going to do it*”. This again raised the suggestion that grades achieved in elective subjects should perhaps not contribute to the overall GPA, with one participant adding, “*if it was GPA neutral I think people would really go explore*”.

#### Skills accumulation

Finally, students considered the skills that could be gained through the Elective Programme. A key example of this is Applied Languages, which participants considered to be a “*real-life skill*” that students may have favoured “*because they think it might be useful to them,*” particularly in relation to travelling and working abroad. Participants indicated a desire for opportunities to progress further in the School of Applied Languages, with one saying, “*I want to be able to speak a language quite proficiently… related more to professional, working life… but didn’t find anything like that*”.

Participants also considered this concept of skills accumulation in relation to the In-Programme electives, saying “*they might do an In-Programme elective to get ahead or to learn something that will be useful in future*” and “*there was the reassurance that an In-Programme elective was going to be vaguely relevant*”.

### Attitudes towards the elective programme

The focus group discussions highlighted a number of other themes that can be classified under attitudes and ideas surrounding the Elective Programme. The general attitudes of participants towards the Elective Programme were varied, with a mixture of enthusiasm and frustration. The broad themes covered include initial impressions, transcript incentives, interface issues and the role of electives in a medical degree.

#### Initial impressions

The majority of participants were not aware of the existence of the UCD Elective Programme before accepting their place in the University, but their initial reactions to it were overwhelmingly positive, with participants saying, “*I was really excited about it when I first found out*”, “*it was a nice surprise*”, “*I thought it was a cool idea*”. When asked if they would have preferred a shorter (five-year) undergraduate degree without free-choice electives, students almost always opted for the longer (six-year) degree with free-choice electives, stating that the alternative would be more “*boring*” and “*far more intense*”. One participant suggested that an advantage of the longer degree programme was “*that extra time gives them the opportunity to get really involved in sports clubs and societies*”.

#### Transcript incentives

The concept of transcript incentives arose frequently in the discussions. Participants indicated a desire for structured electives through which they might be awarded a form of additional minor qualification on their transcript. All participants indicated that this kind of incentive would be a motivating factor, particularly in relation to Applied Languages, saying that it “*would be a great thing to have on your transcript*”, “*I would love* [a structured elective] *in languages*”, “*you would feel like you’re not learning it for no reason*”. Participants emphasised that this incentive would encourage “*progression*” through the difficulty levels as there would be a “*guarantee of continuity throughout*”. Interestingly, the majority of participants were unsure whether this option already existed, having only “*heard about it through hearsay*”. It should be noted that structured electives are currently offered by the UCD School of Social Justice and UCD School of Medicine in the subjects of social justice and radiography respectively, but as one participant pointed out, “*it’s not advertised well at all*”.

#### Interface issues

The interface by which elective options are explored and selected by students is currently in the form of an online tool that is accessed during the registration process at the beginning of each academic year. During the discussions, participants drew attention to a number of issues with this interface, indicating that the tool itself was a hindrance to making optimal elective choices and was described by some participants as “*bizarre*”, “*awkward*”, “*not user-friendly*” and “*extremely difficult to use*”, especially for new students who “*don’t know how to manipulate it properly*” and “*have no idea where to start looking*”. In particular, the first electives that would appear to a student when registering were always the In-Programme electives for that student and more than one participant acknowledged having “*just picked the first two that I saw*”, with one stating “*I thought they were the only ones you could do*” and another adding “*I didn’t know you had to uncheck the box* [to locate non-programme electives]”. Specific issues with the interface included prerequisite issues, oversubscribed electives and difficulty sourcing information.

Participants noted that many of the electives on offer through the online tool were actually unavailable to them due to “*strict prerequisites*”. One participant said “*it was quite frustrating that they were offering electives in this set-up … they were setting up all these barriers*”. Another noted that there were “*hundreds of modules that go on, but you’re excluded from a huge amount of them*”. A similar frustration was expressed in relation to modules from other schools where elective places were limited. Participants mentioned that “*some of the electives are just for certain types of students*” and that modules with limited places for elective students should either “*have more spaces*” or be “*pulled out of the system*”.

Participants also indicated that the protocol for overprescribed electives was problematic as “*you’re left waiting and you don’t know if you got that elective and if you didn’t get into it you’d have nothing left… all the good electives are gone*”, “*if you didn’t get that elective then you were put back into the lottery*”. One participant added that the online tool should list how many “*actual spaces*” remain rather than “*spaces that are already gone*”. However, it was acknowledged by participants that an alternative “*first come first served*” basis would also be “*awkward*” and would likely “*make the system crash*”.

Finally, it was emphasised that the online tool was difficult to navigate in terms of exploring elective options and sourcing the right information. Participants said it was “*definitely hard to find certain electives you had in mind on the system*”; “*I had no idea where to start looking*”; “*the online tool was tiny and you had to keep refreshing it and… go to each school*”, but “*schools are listed as different things, and then apparently, they offer no subjects… it’s just listed in the box*”. Participants struggled with “*refining their search*” saying “*the keywords have to be exact*” and that “*the search mechanism in the site just doesn’t work… you get a page that’s five years old and irrelevant… or the module code has changed or the module no longer exists*”. It was apparent that this lack of confidence in the system was a barrier to students making the best choice, with one participant stating “*I think if it was more easily accessible to find out information about different things, you’d be more likely to do something interesting*” and another adding “*I never even heard of* [a certain elective]; *I would have liked to have done that maybe*”.

On the other hand, several participants did commend the online tool for its explanation of the elective content once it was successfully located, saying “*the descriptions are very clear*”. It would appear then that the problem lies not in the information provided but in the “*formatting*” of the tool, with “*recognition that people couldn’t find interesting electives*”.

#### Electives as part of a medical degree

It became apparent during the discussions that the nature of the UCD undergraduate medical degree programme impacted significantly on both the role and the value of free-choice electives. Since the core material is taught through obligatory modules, the Elective Programme is the only system where choice is available to undergraduate medical students; as one participant put it, “*within medicine we’re quite restricted whereas in other courses you have internal choice*”.

According to another participant, “*for other degree courses I think people are a bit more careful about their elective choice, whereas in medicine it doesn’t shape your degree in any way*”. Others, however, emphasised that the electives were an ideal opportunity to enhance their medical studies through the In-Programme electives and expressed a desire for more In-Programme electives to be available. The majority of participants indicated that they felt ill-prepared for clinical placement, asserting that the core material provided is insufficient, particularly in relation to practical clinical skills. It was suggested that it would be prudent to replace free-choice electives in the latter years with more “*medical focused*” options or “*practical skills sessions*” to rectify this. Indeed, some participants felt that there were too many free-choice electives, saying “*they were nice when we were in stage 1 and 2, but by the time we were choosing our seventh one it was a bit too much*” and “*by the time you choose your seventh you’ve kind of done all of them*”. One participant added “*it’s not my opinion, but I’ve heard a lot of dissatisfaction from peers who think electives are a waste of time*” and another agreed “*I’m a bit ambivalent about the programme; I’ve never quite made up my mind*”.

Overall, however, attitudes to the Elective Programme tended to be positive and participants were eager to voice opinions as to how it could be improved to benefit future students.

## Discussion

Electives have been described as a source of ‘transferable skills’ not just in medicine but across the board in university education [8]. They have been shown to help medical students adapt to change and cope with situations of uncertainty [6], especially as they allow integration into contrasting study courses amid varying cohorts of students [10]. Medical curricula tend to be fact-dense and highly exam-oriented, whereas modules designed by other schools such as liberal arts may be more likely to target alternative cognitive and critical functions [14]. The objectives of undergraduate medical education are characterised, not only as ‘knowledge objectives’ and ‘skills objectives’, but also ‘attitudinal objectives’ [6], the likes of which may be promoted by such an Elective Programme – MacNaughton [10] stated that, with the special-study initiative proposed by the GMC, “*we may... in ten years’ time start producing doctors with a thirst to pursue their education throughout their lives and who are more rounded human beings*”.

With this in mind, it is prudent to determine whether undergraduate medical students in UCD are profiting from the Elective Programme as much as they should be. Certain findings from this research would indicate that the current format of the Elective Programme is not meeting its full potential with respect to the undergraduate medical students in UCD. The evidence for this is as follows.

Quantitative findings from the retrospective analysis demonstrated consistent patterns of elective selection from stage to stage among different year cohorts – namely, that In-Programme electives were invariably the most frequently chosen. This finding may indicate that medical students prefer to choose electives directly related to their course rather than exploring too far outside their ‘comfort zone’. On the other hand, it may be a consequence of the elective selection interface itself, the flaws of which have been discussed, with participants indicating that locating ‘interesting’ electives is difficult with the interface available to them.

Participants in the focus groups emphasised that their elective choices tended to be assessment-based rather than interest-based. It seems counterintuitive that the breadth offered by such a liberal Elective Programme is being negated by the necessity of high grades and exam-based success. Indeed, this counteracts the original philosophy of the Elective Programme, which was to provide such an education as to “*prepare* [students] *to serve society with an awareness of and sensitivity to the cultural, political, economic and social dimensions of their work*” [2]. The suggestion put forward by participants that elective modules be discounted from the GPA calculation is a potential rebuttal to this problem, but is also one that brings further complications, particularly when we consider the Elective Programme as a university-wide scheme. An elective taken by one student may be a core module for another student, thus the grading issue becomes more complex.

Changing the grading mechanism to target this issue may not adjust behaviour in the way it is anticipated. If electives were grade point neutral, it may create a perception that they are less important than the core material and thus generate a two-tier system, causing a proportion of students to disengage. Perhaps the solution lies in changing the culture. The degree programmes are set up to measure students through numbers and grades, and yet the profession, the general public and the august bodies governing the profession readily acknowledge that doctors should be rounded, empathetic and holistic rather than indifferent robotic diagnosticians.

A discrepancy was noted between the data analysis and the qualitative discussions. While participants in the focus groups indicated that choosing ‘easy’ electives was their priority, findings from the retrospective review would seem to contradict this. In-Programme electives were not considered by participants to be ‘easy,’ or to have a low workload, but these were consistently the most chosen. This could be explained by their convenience, accessibility, or perhaps by a feeling among medical students that they are expected to undertake choices that are of relevance to future career. Furthermore, as demonstrated in Fig. 3, elective difficulty levels tended to increase from stage to stage, indicating that either students were deliberately selecting electives of higher difficulty level, or that modules most accessible to them were progressively more difficult. This could indicate that students’ capacity for choice was limited by the accessibility of electives, which could potentially be rectified by adjusting the online tool as referred to previously. However, it could also be an indicator that the assigned elective difficulty levels do not accurately reflect their perceived difficulty or their perceived workload where medical students are concerned.

One of the key findings from the focus groups was that students strongly desired structured elective opportunities. Currently there are structured electives available to UCD undergraduate students in the subjects of radiography and social justice. However, it is clear that these opportunities are not well advertised as none of the students in the focus groups had availed of either, despite the fact that they expressed enthusiasm for the concept. Therefore, in addition to increasing the availability of structured electives – especially in the School of Applied Languages – it is also recommended that awareness of these opportunities be targeted among incoming students.

UCD offers undergraduate medical students a unique opportunity through its Elective Programme and, although it arguably increases the duration of the undergraduate medical degree, participants in the focus groups generally did not see this as a problem, with most indicating a preference for the longer degree programme. In fact, evidence suggests that a six-year degree may be superior to a five-year degree in relation to overall academic outcome [19].

In terms of the broader implications of this study, some key findings are of particular relevance. As discussed previously, the concept that students should design their own learning is one that has gained considerable popularity [6]. An issue that risks going unnoticed however, is the ‘trade-off’ between workload and interest. Focus group discussions revealed that students consider high workload to be a factor that would overrule their interest in a subject if they believed it would impact on their resulting grade. This indicates that students may prioritise good grades over engaging in subjects that interest them, a sacrifice which may hinder the quality of their education. It might be worth exploring whether this is a phenomenon that affects students in other fields to the same extent as medical students.

The significance of the popularity of Applied Language modules should not be overlooked. Applied Languages were consistently the most popular non-programme electives chosen, and students indicated a desire for structured electives in languages as it was believed that learning a foreign language was a skill that would be useful to them. This finding highlights the fact that language electives should perhaps be made more available to medical students in programmes where elective range may be limited. Further research to assess whether this link between medicine and languages is found in other institutions internationally would be very useful.

Participants in focus groups highlighted a number of issues with the way in which free-choice electives were selected in UCD. The recommendation for outside institutions would be to ensure that any interface for elective selection is clear and user-friendly, and that students are made aware of the options and opportunities available to them. A system for providing accurate and detailed information about each elective and its assessment should be put in place so that students don’t just have to rely on ‘hearsay’ or on the advice of their peers as to which electives are the ‘easy’ ones.

Finally, there is a certain significance in the fact that attitudes to the Elective Programme were primarily positive, with the majority of students indicating a preference for the longer, six-year course with provision of free-choice electives rather than a shorter, five-year course without electives. This suggests that despite the issues surrounding assessment and grading, medical students still recognise the inherent value of free-choice electives as part of their learning experience. A comparison between students’ attitudes towards a six-year undergraduate programme with electives and the more internationally implemented model of a four-year undergraduate programme followed by a four-year graduate medical programme might be worth exploring in a future study. Both models offer students the opportunity to branch out into non-medical disciplines and it would be of interest to compare the extent to which this is done, and the benefits or otherwise that are conveyed through such models. It would be of interest, for example, to examine how the priorities of students in an undergraduate pre-medical or liberal arts programme differ from those of their counterparts in an undergraduate medical programme.

This study has a number of limitations. The retrospective data analysis did not take into account the elective choices of advanced entry students (those that did not complete stage 1 due to transfer from other degree courses or prior teaching in international institutions). It did not take into account student demographics other than gender. The qualitative study involved only a small cohort of student participants (*n* = 16) who volunteered to take part during out-of-semester time and thus may not be representative of the undergraduate medical student body. Although the repetition of key ideas and the consistency between focus groups suggested a substantial degree of saturation, the authors felt that the sample size was smaller than desired. Time-restrictions and low numbers of respondents limited the number of focus groups that could be held.

With the exception of the pilot focus group, the sample was deliberately restricted to stage 4 medical students, whose views may vary from those of students in earlier stages in the programme. Future studies may benefit from seeking the views of students in earlier stages as a comparison.

The participants in focus groups were current medical students and therefore were not included in the retrospective analysis cohort. The study did not examine the elective choices of students outside the School of Medicine.

Institution-specific recommendations from this study are as follows. The online tool for elective selection should undergo review and adaptation to make it more user-friendly and to improve accessibility. Structured electives should be advertised more prominently and should be offered in more subjects, including languages. Students should be incentivised to take electives in subjects that interest them rather than opting for electives that they believe will get them a better grade. To achieve this, it is recommended that a thorough review of the assessment strategy for both core and elective modules be carried out with the intention of eliminating the risk associated with choosing challenging electives. One possible solution lies in excluding electives from the cumulative degree GPA, without making them GPA neutral.

## Conclusion

The paper presents an analysis of the choices that medical students make when selecting subjects for the Elective Programme with a view to exploring the factors that medical students prioritise when designing their own learning.

UCD medical students were found to have a preference towards In-Programme modules, which are aligned to medical themes and designed to complement the core material. This could be explained by a number of factors, but is believed to be a combination of convenience, interest in the In-Programme subject material and belief that the skills learned would be applicable in the future. Applied Language electives were also found to be widely popular, perhaps because of their practical application, but also due to the perceived fun, interactive nature of the small group classes. Patterns of elective selection tended to be relatively consistent in terms of stage, year cohort and gender, indicating that this information may be manipulated to plan for future elective provision both within UCD and in other institutions that should choose to replicate its Elective Programme.

Students choose electives at least partly on the basis of their perceived workload and assessment strategies. There exists a trade-off between academic success (that is, achieving a high grade) and the desire to study interesting or challenging subject matter. Strategies recommended to alleviate this trade-off could include making free-choice electives GPA neutral, altering the assessment strategy of those electives perceived to be more challenging in order to lighten the workload at key points in the semester or providing transcript incentives such as structured electives – that is, displaying pedagogically linked electives as a minor qualification within the major degree, something that students indicated would be a positive motivating factor.

A major recommendation for medical programmes offering electives or options would be to ensure consistent accessibility, level of difficulty and grading practices if the aim is to offer options aligned to both the student’s academic profile and personal interests.

On a practical level, the focus group discussions highlighted the importance of having a suitable elective selection process and of promoting awareness of the diverse opportunities available in order to optimise the learning experience of each student.

These findings may be used by institutions both nationally and internationally to guide the development or enhancement of similar elective programmes, liberal pre-medical programmes or even optional modules within medical programmes.

## Abbreviations

AMEE: Association for Medical Education in Europe
GMC: General Medical Council
GPA: Grade Point Average
UCD: University College Dublin
